# P-1994. Faster than Culture. Use of 16/ITS Next-Generation Sequencing for Rapid Identification of Pathogens Directly from Brain/Cerebellar Samples

**DOI:** 10.1093/ofid/ofaf695.2159

**Published:** 2026-01-11

**Authors:** Jose Alexander

**Affiliations:** Advent Health Central Florida, Orlando, FL

## Abstract

**Background:**

It is of critical importance to accurately and timely provide organism identification for brain and cerebellar abscess. Rapid and appropriate identification of causative organisms is crucial for guiding effective treatment strategies and improving patient outcomes. However, traditional culture-based diagnostic methods often face significant challenges, including low sensitivity, extended turnaround times, and inability to detect fastidious or anaerobic pathogens, leading to delayed or inappropriate treatment. Next-generation sequencing (NGS) can detect a wide range of organisms, including bacteria, mycobacteria, and fungi, even in culture-negative cases. In this study, we compare the result of NGS performed directly on brain aspirate/tissue samples against traditional culture.

**Methods:**

A total of 11 tissues or swabs from brain or cerebellar cortex were sent for 16S/ITS NGS (MicroGenDX) testing by AdventHealth Orlando from June 2023 to September 2024. This test is based on a qPCR (Level 1) that includes antimicrobial resistance genes and 16S/ITS (Level 2) with a 3-day turnaround time. All samples were cultured in aerobic and anaerobic conditions in-house on tissue/surgical culture and held for 5 days.

**Results:**

7/11 (63.3%) samples were positive by 16S/ITS. Four *Streptococcus intermedius*, one *Cutibacterium acnes*, one *Staphylococcus aureus*, and one *Klebsiella pneumoniae*. 5/11 (45.6%) samples were also positive on culture for two *S. intermedium*, *S. aureus,* and *K. pneumoniae*. Resistance markers were performed by PCR along with 16S/ITS. *mecA* gene for *S. aureus* and *CTX-M, KPC* and *NDM* for *K. pneumoniae* were not detected.

**Conclusion:**

16S/ITS NGS identified an infectious organism in 63.3% (7/11) of brain samples directly from the specimen, 45.6% (5/11) of the samples were also positive on culture. The absences of *mecA*, *CTX-M, KPC,* and *NDM* were confirmed by phenotypical susceptibility of the *S. aureus* and *K. pneumoniae.* NGS turnaround time of 3 days returned a clinically significant and correlating result faster when compared to culture, with an average of 5.5 days. For rapid organism identification, NGS seamlessly complements culture, offering faster and clinically actionable results for brain samples enabling prompt antimicrobial intervention.

**Disclosures:**

All Authors: No reported disclosures

